# Lipidomics and genomics of *Mycobacterium tuberculosis* reveal lineage-specific trends in mycolic acid biosynthesis

**DOI:** 10.1002/mbo3.193

**Published:** 2014-09-19

**Authors:** Damien Portevin, Sudarkodi Sukumar, Mireia Coscolla, Guanghou Shui, Bowen Li, Xue Li Guan, Anne K Bendt, Douglas Young, Sebastien Gagneux, Markus R Wenk

**Affiliations:** 1 Mycobacterial Division Research, NIMR, MRCNW71AA, London, United Kingdom; 2 Department of Medical Parasitology and Infection Biology, Swiss TPH4002, Basel, Switzerland; 3 University of Basel4002, Basel, Switzerland; 4 Singapore-MIT Alliance, National University of Singapore117576, Singapore; 5 State Key Laboratory of Molecular Developmental Biology, Institute of Genetics and Developmental Biology, Chinese Academy of SciencesBeijing, 100101, China; 6 Department of Computer Science, National University of Singapore117417, Singapore; 7 Centre for Life Sciences, National University of Singapore117456, Singapore; 8 Department of Biochemistry, Yong Loo Lin School of Medicine, National University of Singapore117456, Singapore; 9 Department of Biological Sciences, National University of Singapore117543, Singapore

**Keywords:** Genomics, lipidomics, mycolic acid, phylogenetics, tuberculosis

## Abstract

Mycolic acids (MAs) are *α*-alkyl, *β*-hydroxy long-chain fatty acids found in abundance in the cell envelope of the *Mycobacterium tuberculosis* complex (MTBC). MAs form an efficient permeability barrier, modulate host innate immune responses, and are the targets of several anti-tuberculosis drugs. Using mass spectrometry, we measured the relative abundance of 80 MA species across 36 clinical isolates of MTBC covering four major phylogenetic lineages. We found significant variations in the MA patterns between different MTBC strains and lineages. MA patterns of “ancient” lineages contrasted those from “modern” lineages, with a lower representation of alpha-mycolates among Lineage 6 strains and an inversion of the methoxy: keto-mycolates ratio in Lineage 1 strains. By interrogating the whole genome sequences of these MTBC strains, we identified relevant single-nucleotide polymorphisms that may sustain the lineage-specific MA patterns. Our results show that the strain genetic background influences MA metabolism and suggests that strain diversity should be considered in the development of new anti-tuberculosis drugs that target MA synthesis.

## Introduction

Mycolic acids (MAs) are *α*-alkyl, *β*-hydroxy long-chain fatty acids found exclusively in the cell envelope of *Corynebacteriales*, an order that includes the etiological agent of tuberculosis: *Mycobacterium tuberculosis* complex (MTBC). MAs constitute the most abundant cell wall lipid in MTBC, that reaches up to 60% of the cell envelope dry weight (Yassin 2011). MTBC produces three major classes of MAs derived from different enzymatic modifications of the main fatty acid meromycolic chain: (1) alpha-, (2) methoxy- and (3) keto-mycolates. MA profiles within individual species of mycobacteria are conserved (Watanabe et al. 2001). Indeed, a mass spectrometry (MS)-based approach was used recently to successfully discriminate MTBC from nontuberculous mycobacteria (Shui et al. 2012). However, a comprehensive analysis of MA profiles among phylogenetically distant strains of MTBC has not yet been reported.

Substantial genetic diversity has been described among the MTBC strains prevalent in different parts of the world (Gagneux et al. 2006). The human-associated MTBC consists of seven main phylogenetic lineages (Comas et al. 2013), and increasing evidence suggests that this phylogenetic diversity has important phenotypic consequences (Coscolla and Gagneux 2010). For example, MTBC lineages have been shown to differ in their rate of progression to active disease or in their propensities to cause extra-pulmonary disease (Caws et al. 2008; de Jong et al. 2008). Transmission studies have shown that some strains belonging to the so-called Beijing family are more likely to cause secondary cases (Sreevatsan et al. 1997). However, the bacterial factors involved remain poorly understood. MTBC is known to produce wide varieties of complex lipids, which is also reflected in the large number of genes that are predicted to be involved in lipid metabolism (Cole et al. 1998). MTBC strain-specific lipid profiles, notably in the Beijing strain family, have been associated with increased transmission and drug resistance (Reed et al. 2007; Ford et al. 2013). Moreover, a number of in vitro and in vivo studies have reported evidence of variable inflammatory capacities among MTBC strains (Aguilar et al. 2010; Wang et al. 2010) and that both glycolipid biosynthesis and subtle chemical modifications carried by different MA subspecies can substantially affect macrophage cytokine responses and virulence in mice infection models (Reed et al. 2004; Rao et al. 2005; Barkan et al. 2012). Vander Beken et al. (2011) demonstrated the role of these functional group modifications mediated by MAs in eliciting unique inflammatory patterns. Therefore, specific changes in MA profiles are likely to play a role in the variety of inflammatory responses elicited by different clinical strains of MTBC (Portevin et al. 2011), potentially in a toll-like receptor 2 (TLR-2)-dependent manner (Sequeira et al. 2014). MA modifications are also thought to contribute to resistance against oxidative stress (Yuan et al. 1995), suggesting that variations in MA composition between strains may confer altered levels of protection from the hostile environment encountered within macrophages. Finally, MAs constitute a core lipid of the MTBC cell envelope and a physical hydrophobic barrier to antibiotics, as demonstrated by mutants affected in MA metabolism (Singh et al. 2005). Yet, the variability of MA cell wall composition has not been studied in a comprehensive way that integrates MTBC phylogenetic diversity.

In this study, we used lipidomics to characterize the variation in MA profiles across different MTBC clinical strains belonging to four major phylogenetic lineages. By integrating a comprehensive biochemical characterization of MA metabolism with full genome data, we found that the MTBC lineages differed significantly in their MA profiles. Moreover, we predict a functional impact of several single-nucleotide polymorphisms within the MA pathway that could sustain the different patterns of MAs.

## Experimental Procedures

### Mycobacterial cultures and isolation of MAs

Mycobacterial cultures were expanded following a single colony-forming unit isolation step and strain typing analysis. Stationary phase pre-cultures of mycobacteria performed in Middlebrook 7H9 medium with ADC supplement (BD Biosciences, Sparks, MD 21152, USA), 0.05% Tween-80 (Sigma-Aldrich, St. Louis, MO) and 40 mmol/L sodium pyruvate were diluted with 100 volumes of the same medium in the absence of detergent and incubated under static conditions for 10 days at 37°C. Mycobacterial flakes were recovered by centrifugation and inactivated with 750 *μ*L of chloroform/methanol (2:1). MAs were extracted as described by Shui et al. (2012).

### MRM-based MS for relative quantification of MAs

The extracted MAs were resuspended in chloroform/methanol (1:1). The MA extracts were diluted appropriately using chloroform/methanol (1:1) containing 2% piperidine at 300 mmol/L and subjected to direct infusion at a flow rate of 15 *μ*L/min. The MAs were measured in the negative Electrospray Ionisation (ESI) mode using a list of multiple reaction monitoring (MRM) transitions established as described by Shui et al. (2012). A total of 80 MRM transitions were used to quantify major MTB MA species. The individual MA intensities were median-fold normalized, that is, relative abundance is a ratio of a specific signal intensity over median of all MA intensities in a single strain. Representation was obtained as a percentage of a specific MRM intensity toward the sum of all signals for each strain independently. Normalized data including median intensities used for normalization are provided in Table S1.

### Illumina sequencing, mapping and SNP calling

Mycobacterial DNA was isolated as previously described (Ausubel et al. 1987) and libraries generated using standard kits from Illumina (San Diego, CA). Sequences were generated using HiSeq 2000 single-read 50 bases and MiSeq 2 × 250 bases. We used BWA to map Illumina reads from the eight genome sequences newly published in this study (study accession number PRJEB5148) and 27 genomes published previously (Comas et al. 2010) against the MTBC reference genome (Comas et al. 2013). The sequencing depth for N0081 did not allow enough coverage for the SNP calling and this strain could not be incorporated into subsequent phylogenetic analyses. BWA outputs were analyzed with *S*-adenosyl-l-methionine (SAM) tools (Li et al. 2009) and after SNP calling and filtering as in (47), we kept 11,531 high-confidence variable positions for downstream analysis.

### Phylogenetic analysis

Maximum likelihood phylogenies were obtained using phyML (Guindon et al. 2010) and general time-reversible (GTR) model and specifying *M. canettii* as the outgroup. Branch robustness was assessed through bootstrapping (1000 pseudo-replicates).

### SIFT and conserved domain prediction analysis

SIFT prediction was performed using the online tool (http://sift.jcvi.org/www/SIFT_seq_submit2.html) with a median conservation of sequences of three to filter biased predictions and 90% identity filtering. Protein domains were identified using the Conserved Domains search engine of the NCBI.

### Statistical analysis

Two-tailed Mann–Whitney tests were performed using GraphPad prism (version 4.03, San Diego, CA, USA). Heat-map was generated using PermutMatrix (version 1.9.3; Montpellier Cedex 5, France). Principal component analyses were conducted using Paleontological Statistics Software package for education and data analysis, version 2.17c (Hammer, Ø. et al., 2001).

## Results

### MA genealogy mirrors the genome-based MTBC phylogeny

**Figure 1:**
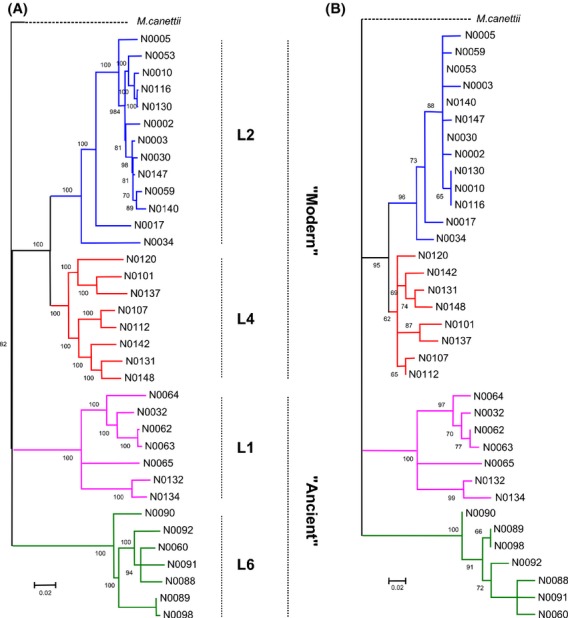
Phylogenomic analyses of selected*Mycobacterium tuberculosis* complex (MTBC) clinical isolates. Maximum likelihood phylogeny of 35 MTBC strains used for this study based on single-nucleotide polymorphisms (SNPs) extracted from (A) full genome data (e.g. 2795 synonymous SNPs) and (B) polymorphisms in genes related to the mycolic acid pathway (e.g. 98 nonsynonymous SNPs). Bootstrap values indicating node support are indicated. Four lineages of *M. tuberculosis* strains affecting humans are represented and highlighted with the same color code across the study.

A recent analysis suggested strong purifying selection in genes important for the physiology of MTBC (Pepperell et al. 2013). Therefore, we would expect that essential genes, such as those involved in MA metabolism, should be conserved. However, because MA metabolism is also the primary target of isoniazid and ethionamide, two main antibiotics currently used to treat tuberculosis, the genes involved in MA metabolism may be under diversifying selection. To explore the extent to which the MA machinery might differ across the different lineages of MTBC, we first studied the genome sequences of 35 clinical strains from four main MTBC lineages, which we used subsequently for MA profiling (see below). These 35 strains included representatives of the so-called “modern” and “ancient” phylogenetic lineages, the latter branching earlier in the evolution of MTBC (Brosch et al. 2002; Hershberg et al. 2008). Figure 1A displays the phylogeny among strains derived from neutral DNA sequence variation (synonymous single-nucleotide polymorphisms: sSNPs), which shows the same relationships among lineages as the genome-based MTBC phylogenies described previously (Comas et al. 2009).

To specifically explore the phylogenetic signal contained in genes related to MA metabolism, we extracted from the literature a list of 62 genes coding for proteins demonstrated or proposed to be involved in MA anabolism (Takayama et al. 2005), (Kastrinsky et al., 2010) (Table S3). We then built a phylogenetic tree using only the mutations that should have the greatest impact on MA metabolism; that is, the nonsynonymous single-nucleotide polymorphisms (nsSNPs) restricted to this pathway (Fig. 1B, SNP list provided as Table S2). The phylogeny resulting from nsSNPs within the MA pathway mirrored the phylogeny inferred from full genome data. This suggests that the genetic diversity present within the MA pathway contains most of the information necessary to reconstruct the phylogenetic relationships among the MTBC strains studied here, and as a consequence, MTBC lineages are likely to differ in their MA metabolism. To test this prediction, we used a lipidomics approach to define the MA profiles of these phylogenetically distant MTBC clinical strains.

### MA profiling distinguishes MTBC lineages

**Figure 2:**
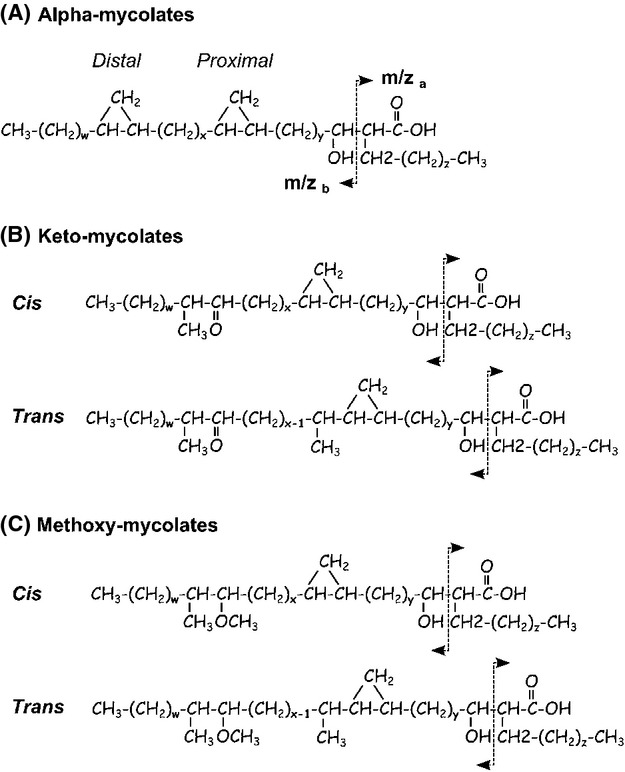
Structure and mass spectrometry fragmentation of the mycolic acid species present in *Mycobacterium tuberculosis* complex (MTBC). (A) Alpha-mycolates harbor both distal and proximal cyclopropane modifications on their meromycolic chain. (B) Keto-mycolates have a cyclopropane group in the proximal position and a ketone in the distal position of the meromycolic chain. (C) Methoxy-mycolates harbor a cyclopropane group in the proximal and a methoxy group in the distal positions of the meromycolic chain. *Cis*/*trans* stereoisomers of keto- and methoxy-mycolic acids (MAs) are distinguished according to the presence (*trans*) or not (*cis*) of a methyl group in alpha of the proximal cyclopropane function. MA species vary in length (72 < *x* + *y* + *z* < 89). The arrows indicate the major fragmentation pattern used for mass spectrometry identification of individual species by multiple reaction monitoring (Shui et al. 2012).

**Figure 3:**
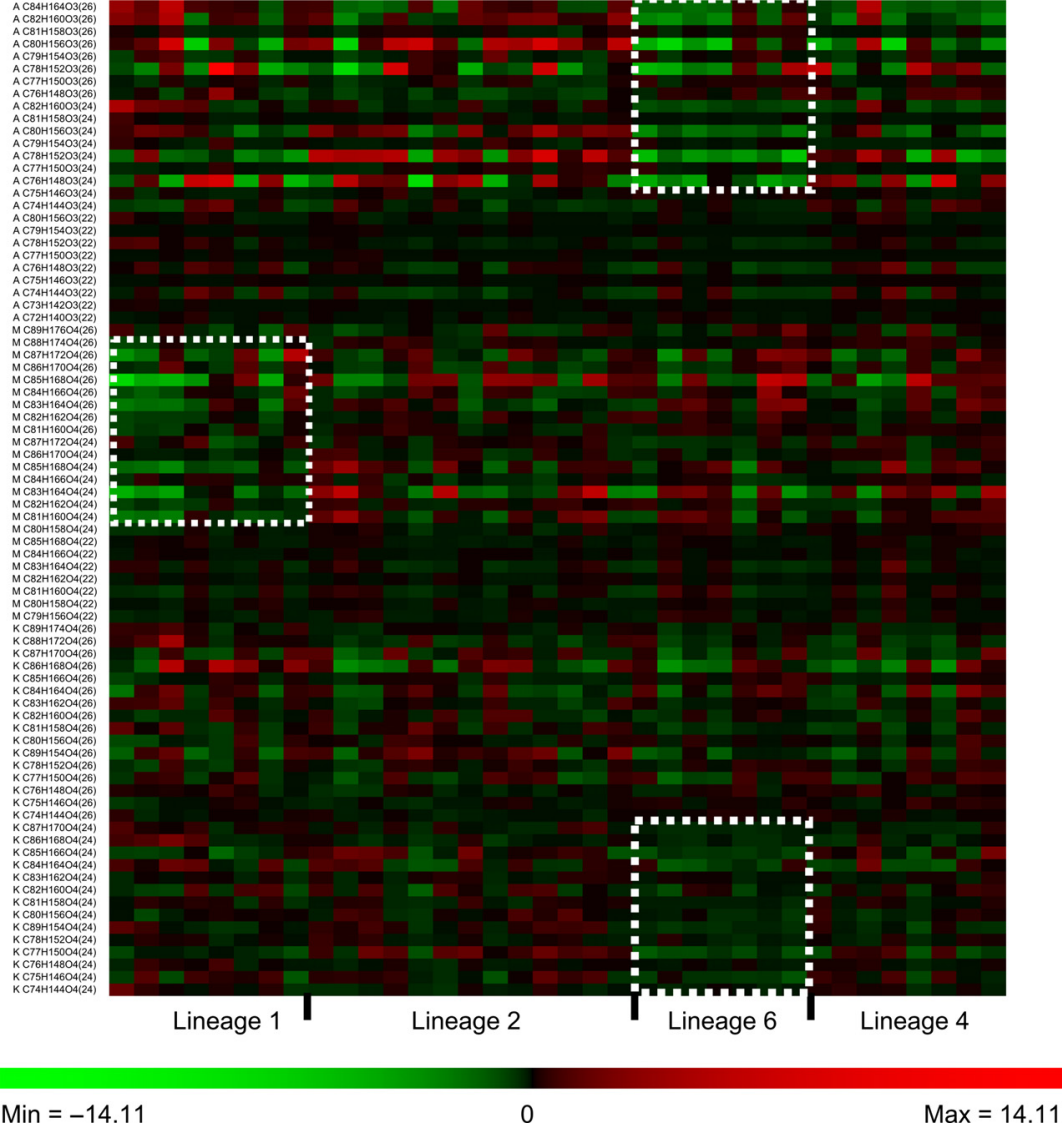
Mean row centered heat-map repre-sentation of median normalized intensities of individual mycolic acid molecular species across 36 strains and four lineages of *Mycobacterium tuberculosis* complex. Appearing trends that were visually evident are highlighted with dashed squares (Raw data provided as Table S1).

**Figure 4:**
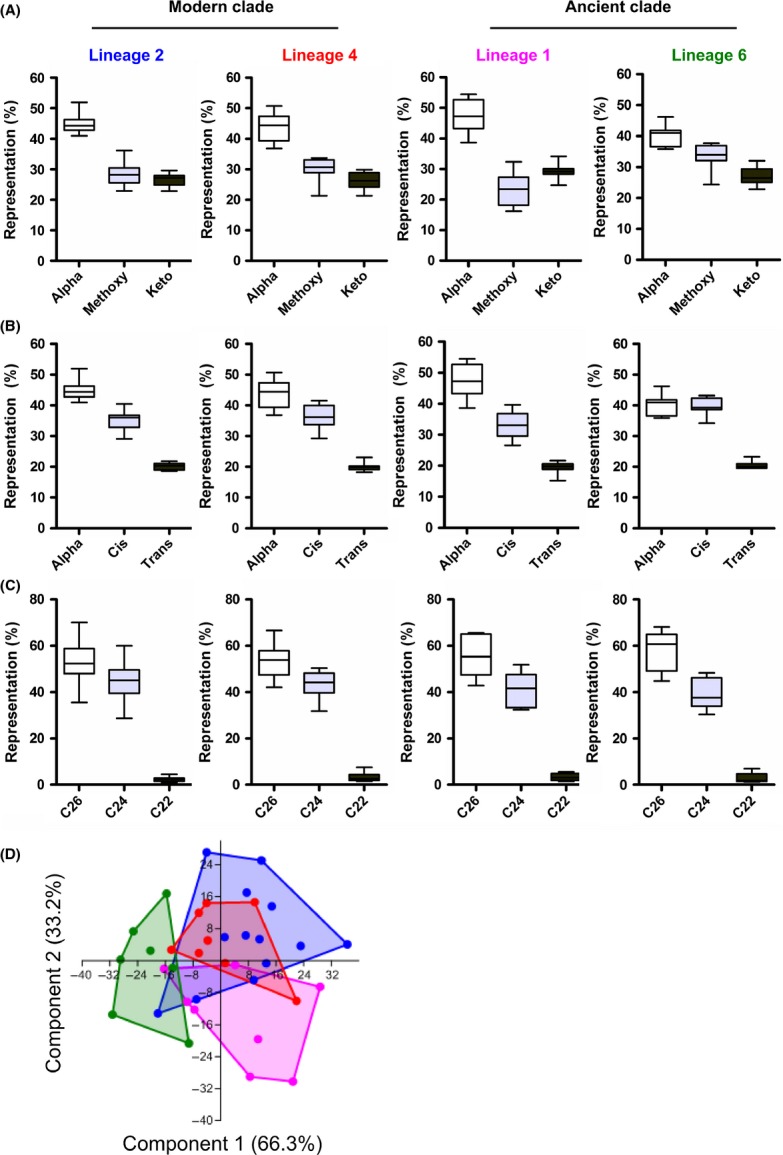
Mycolic acid profiling of the different *Mycobacterium tuberculosis* complex (MTBC lineages. (A) Representation of alpha-, methoxy- and keto-mycolic acids (MAs) across the different MTBC lineages. (B) Representation of alpha-, *cis*- and *trans*-isomers of oxygenated MAs across the different MTBC lineages. (C) Representation of MAs species classified according to the length of the fatty acid chain in alpha of the meromycolic acid chain (“alpha-branch”) across the different MTBC lineages. (D) Scatterplot derived from principal component analysis of strain-specific mycolic acid profiles, showing that component one and component two covering 99.5% of sample variance discriminate Lineages 1 and 6, respectively, whereas “modern” Lineages 2 and 4 mostly overlap with each other.

MAs in MTBC comprise molecules that vary in length and by the presence or absence of specific chemical groups created by various enzymatic modifications of the meromycolic chain precursor (Fig. 2) (Takayama et al. 2005). We used MS (Shui et al. 2012) to measure 80 MA species synthesized by our set of MTBC clinical isolates. Each strain was tested once and considered as a biological replicate within its respective lineage for downstream analysis. Arrows in Figure 2 indicate the actual fragmentation pattern of precursor/product ion pairs we used to discriminate among the different MA species. We observed substantial variability in the relative quantities of individual MA species between the different strains using a mean-row centered heat-map (Fig. 3). It is noteworthy that individual strain diversity in MA profiles within the same lineages could be observed. Although, since analysis of nsSNPs in the MA pathway discriminated between different MTBC lineages, we first compared the abundance of each major structural MA variant among these lineages (Fig. 4A). Earlier studies described alpha-MAs as the most prominent MA species in MTBC, followed by methoxy-MAs and keto-MAs (Watanabe et al. 2001). We observed a similar pattern for the two “modern” lineages (i.e. Lineages 2 and 4). However, the proportion of methoxy- and keto-MAs in the “ancient” Lineage 1 and the proportion of alpha- and methoxy-MAs in Lineage 6 differed (Fig. 4A).

Of note, the stereochemistry of the first or “proximal” cyclopropane group of oxygenated mycolates (keto- and methoxy-MAs) can be extrapolated from the MS data, as alpha-methyl-*trans*-cyclopropane-MAs have a methyl group that is missing from the equivalent *cis*-MA species (Fig. 2). Indeed, *cis*-keto-MAs harboring a methyl group in alpha of the keto function and a *cis* conformation of the proximal cyclopropane group should have an even number of carbon atoms, whereas equivalent *trans*-keto-MA species harboring an additional methyl group in alpha of the proximal cyclopropane should have an odd number of carbons in total. However, equivalent species of *cis*-methoxy-MAs and *trans*-methoxy-MAs should have, respectively, odd and even numbers of carbon atoms as a result of the presence of the methoxy group. Based on this logic, we calculated the distribution of alpha-MAs and the *cis*/*trans* stereoisomers within the oxygenated-MA species (Fig. 4B). After alpha-MAs, *cis*-oxygenated MAs constituted the most abundant species followed by *trans*-oxygenated MAs; this was evident for all lineages except Lineage 6, which showed a similar proportion of alpha- and *cis*-oxygenated MAs.

Finally, we studied the length of the fatty acid *α*-ramification (*α*-branch) across the different lineages independent of the functional group modifications carried by the meromycolic acid chain. Overall, the vast majority of MAs were ramified with either C26- or C24- and, to a lesser extent, with C22-fatty acids (Fig. 4C) as their *α*-branch.

**Figure 5:**
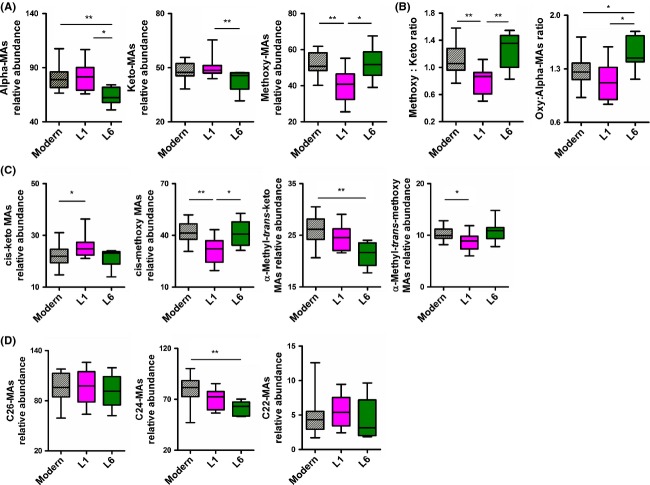
Profiling of mycolic acid (MA) species, isomers and alpha-branch length across the different *Mycobacterium tuberculosis* complex (MTBC) lineages. (A) Relative quantification of alpha-, keto- and methoxy-MA species between “modern”, Lineage 1 (L1) and 6 (L6) of MTBC. (B) Comparison across the different MTBC lineages of the ratio of methoxy- to keto-MAs and the ratio of oxygenated (e.g., methoxy-MAs plus keto-MAs) to alpha-MAs. (C) Relative quantification of *cis* and alpha-methyl-*trans* isomers within keto-MAs and methoxy-MAs across the different MTBC lineages. (D) Relative quantification of MAs species classified according to the length of the *α*-branch MAs and across the different MTBC lineages (two-tailed Mann–Whitney test, *P < 0.05, **P < 0.01).

A principal component analysis based on median-fold normalized intensities of the MA species accounted for 99.5% of variance within the first two components. As depicted in Figure 4D, Lineage 1 and Lineage 6 strains formed independent clusters, whereas strains belonging to the “modern” lineages were indistinguishable from each other (Fig. 4D). Hence, Lineage 2 and 4 strains were subsequently grouped as “modern” for statistical comparisons with strains from Lineages 1 and 6. Relative intensity plots revealed that alpha-MAs in Lineage 6 and methoxy-MAs in Lineage 1 strains were statistically significantly less-represented in comparison with the other lineages (Fig. 5A). Computing the ratios between methoxy- and keto-MAs revealed a statistically significant inversion in Lineage 1 (median: 0.87) as compared with the “modern” lineages (median: 1.06, *P* = 0.004) and Lineage 6 (median: 1.36, *P* = 0.009) (Fig. 5B). Similarly, we calculated the ratios between oxygenated-MAs (methoxy + keto) and alpha-MAs and found a statistically significant increase for Lineage 6 (median: 1.44) compared with the “modern” lineages (median: 1.266, *P* = 0.017) and Lineage 1 strains (median: 1.118, *P* = 0.02) (Fig. 5B).

As for *cis*/*trans*-oxygenated MA isomer species, despite the overall similar representation of *cis*-MAs in Lineage 1 strains compared with the other lineages (Fig. 4B), a detailed analyses revealed that the amount of *cis*-keto-MAs in these strains was significantly higher than in the “modern” lineages strains (*P* = 0.04); this counter-balanced the significant reduction in the *cis*-methoxy-MAs when compared with the “modern” and Lineage 6 strains (*P* = 0.002 and *P* = 0.04, respectively, Fig. 5B). We also observed a lower representation of alpha-methyl-*trans*-keto-MAs in Lineage 6 and of alpha-methyl-*trans*-methoxy MAs in Lineage 1 as compared with the “modern” strains (*P* = 0.002 and *P* = 0.02, respectively). Regarding the length of the fatty acid chain ramification in alpha of the meromycolic acid chain, we found that C24-MA species were significantly less represented in Lineage 6 strains and that this was particularly marked when compared with the “modern” strains (*P* = 0.002). Noteworthy, and as suggested by the principal component analysis, we found no significant differences for any of the variables described above between the two “modern” lineages (Fig. S1).

### nsSNPs predicted to affect protein function accumulated among “ancient” lineages

**Figure 6:**
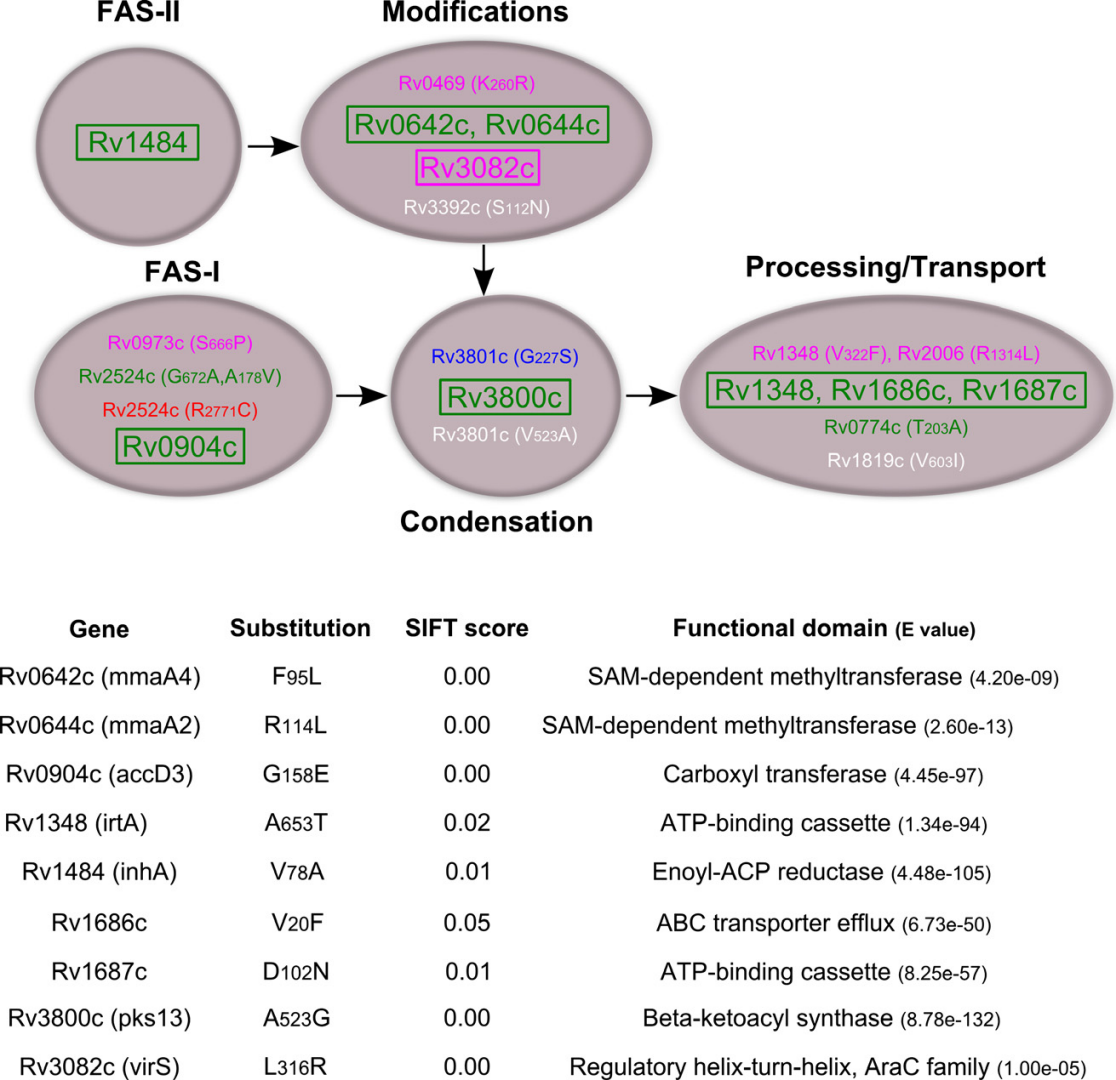
Lineage-specific nonsynonymous single-nucleotide polymorphisms (nsSNPs) from “ancient” lineages are predicted deleterious. nsSNPs specific to each *Mycobacterium tuberculosis* complex (MTBC) lineage were extracted and the related genes listed and grouped according to their involvement in the different step of the metabolic pathway of mycolic acid biosynthesis (white color code was used for SNPs shared by the two “modern” lineages). The amino acid substitutions were subjected to SIFT algorithm to predict loss of function based on the degree of conservation of amino acid residues in sequence alignments derived from closely related sequences (Sim et al. 2012). Genes highlighted in boxes presented substitutions with a statistically significant probability to affect protein function (*P* < 0.05) whereas others were predicted to be tolerated. Gene names, amino acid substitution, SIFT score as well as the predicted domain affected by the mutation are presented for deleterious substitutions.

Nonsynonymous SNPs among the MA pathway were extracted from full genome sequences and listed in Table S2. We identified 97 SNPs across all four lineages, and almost 50% of them were singletons; that is, only detected in one strain. About 30% of these polymorphisms were shared by two strains or more within the same lineage. We identified 17 nsSNPs present exclusively in one of the four MTB lineages, and three nsSNPs shared by the two “modern” lineages. Only one lineage-specific nsSNP was found in each of Lineage 2 and 4, whereas five and 11 nsSNPs were detected in Lineage 1 and 6, respectively. The genes were grouped according to their involvement in the MA metabolism pathway and depicted in Figure 6. We then investigated the predicted functional effect that individual nsSNPs, and therefore amino acid substitution, could bring to the related protein using SIFT prediction algorithm (Sim et al. 2012). When the amino acid substitution was predicted to have a significant effect on protein function (*P* < 0.05), the respective gene was highlighted (Fig. 6). Interestingly, nine of the 20 nsSNPs that were predicted to affect protein function belonged to “ancient” strains but none to the “modern” lineages.

## Discussion

Combining lipidomic and genomic approaches, we present a comprehensive analysis of MA profiles among phylogenetically distant MTBC strains. Our study highlighted trends in MA metabolism that are shared by strains from same phylogenetic lineages. Noteworthy, substantial intralineage diversity could also be observed at the metabolic level, which is consistent with the high number of singleton SNPs and SNPs present in two or more strains but not in the entire lineage. Despite this intralineage diversity, we observed significant differences in the proportion of the different structural variants of MA species between phylogenetic lineages of MTBC. The metabolism of oxygenated MAs was found to differ especially among “ancient” lineages compared with “modern” ones. These changes are of particular importance in the context of tuberculosis treatment, since a change in the methoxy-MA to keto-MA ratio could lead to an altered baseline susceptibility to anti-mycobacterial drugs in the absence of any drug resistance mechanisms (Sambandan et al. 2013). Interestingly, the most significant MA differences were observed within Lineage 1 and 6 lineages, which is consistent with the higher number of predicted functional mutations in genes involved in MA metabolism detected in these “ancient” lineages. Changes in the production of oxygenated-MAs are likely linked to enzymatic reactions involved in the modifications of the meromycolic chain. All Lineage 1 strains showed a mutation in *umaA1*, a recently described SAM-dependent methyltransferase (Meena et al. 2013), but the amino acid substitution present in this strain is unlikely to affect protein function, according to our bioinformatic prediction. However, all Lineage 1 strains also showed an L316R substitution in Rv3082c (*virS*) predicted to affect protein function. Interestingly, the deletion of *virS* in MTBC was shown to lead to a 50% decrease in MA anabolism. Moreover, the mutated strain exhibited a fourfold higher sensitivity to rifampicin and ciprofloxacin, and a twofold higher sensitivity to isoniazid in comparison with the parental strain (Singh et al. 2005). VirS inhibits its own transcription and the L316R substitution affecting its DNA-binding domain is indeed functional, as demonstrated recently by its constitutively enhanced expression in a representative Lineage 1 strain (Rose et al. 2013). By extension, with a *virS* mutant phenotype, and in the absence of any compensatory mechanism, Lineage 1 strains should be generally more susceptible to antibiotics. Supporting this hypothesis, a recent study reported that the concentration of rifampicin required to kill 100% of cells was eight times lower in a Lineage 1 strain when compared to a Lineage 2 strain (de Steenwinkel et al. 2012).

Lineage 2 strains, and the Beijing subtype in particular, are thought to be important drivers of drug resistance in MTBC (Hanekom et al. 2011). The proposed underlying mechanisms include greater transmissibility (Yang et al. 2012) and a higher intrinsic mutation rate (Ford et al. 2013). Bacterial mutation rates reflect the sum of mutations occurring during DNA replication and those accumulating as a consequence of stress responses in nonreplicating bacteria (Martinez and Baquero 2000). Therefore, subtle changes in antibiotic minimal inhibitory concentrations (MICs) due to lineage-specific metabolism of MAs could affect the population dynamics by increasing the population size dividing or persisting in a nonreplicating state (Müller et al. 2013). For example, variations in MA content as a result of a *virS* inactivation could affect cell wall permeability and hence decrease drug tolerance. Generally, we observed that MA patterns differed substantially between individual MTBC strains, suggesting an even broader range in drug tolerance across individual MTBC strains. Lineage- or strain-specific variability in antibiotic permeability linked to MA content would also have significant clinical implications, given that strain genotyping could be implemented in the future in the frame of a personalized approach to tuberculosis treatment (Müller et al. 2013).

We also identified two nsSNPs specific to Lineage 6 with a predicted functional effect in two enzymes involved in the modifications of the meromycolic acid chain: Rv0642c (*mmaA4*) and Rv0644c (*mmaA2*). The enzyme encoded by *mmaA2* has been shown to introduce the distal cyclopropane modification of alpha-MAs (Glickman 2003). The potential functional effect of MmaA2 due to R114L substitution in Lineage 6 strains would be consistent with the reduced proportion of alpha-mycolates observed here. However, we did not note a reduction in *cis*-methoxy-MAs as indicated by the *mmaA2* null mutant, which suggests a partial loss of function, specific to the distal cyclopropane modification. Deletion of *hma* (*mmaA4*) in MTBC demonstrated the involvement of the encoded enzyme in the metabolism of the precursor for both oxygenated-MA species (Dubnau et al. 2000). Since the F95L substitution, which affects *mmaA4* in all Lineage 6 strains, was also predicted to affect protein function, this would imply that production of oxygenated-MAs is also affected in these strains. Together with a reduction in alpha-mycolates, this could contribute to the significant increase in the ratio between oxygenated and alpha-mycolates that we observed in Lineage 6 strains. Importantly, two other predicted functional substitutions in *pks13* (A523G) and in *inhA* (V78A) were found as a specific trait of Lineage 6 strains. Pks13 activity is essential for the survival of mycobacteria (Portevin et al. 2004), and InhA is targeted by the anti-TB drugs, isoniazid and ethionamide. Taken together, several steps of MA metabolism could be affected in Lineage 6 strains. Interestingly, the growth rate of this lineage has recently been shown to be significantly reduced when compared with “modern” MTBC strains, and this difference was suggested to be partly associated with a higher mutation rate in lipid transport-associated genes (Gehre et al. 2013). Our analysis suggests that suboptimal MA metabolism could also contribute to the observed growth rate reduction in Lineage 6.

All predicted functional mutations occurred in the two “ancient” lineages studied here. Even though the amino acid substitution due to other nsSNPs may not affect protein function, they could modify the degree of interaction with other enzymatic partners, as demonstrated by the existence of various multi-protein complexes required for the biosynthesis of MAs (Veyron-Churlet et al. 2005). Nevertheless, when considering the rate of progression to disease, “modern” lineages might be outcompeting “ancient” lineages strains, most notably *M. africanum* (i.e. MTBC Lineage 5 and 6) (de Jong et al. 2008; de Jong et al. 2010). We showed that *M. africanum* – Lineage 6 strains in particular, accumulated functional nsSNPs that should affect the overall metabolism of MA. Because MAs are a major and essential component of the mycobacterial cell wall, this could in turn lead to the lower pathogenicity of these “ancient” strains.

Regulatory mechanisms, including noncoding RNAs (Arnvig et al. 2011) or epigenetic mechanisms (Shell et al. 2013), could also be involved in the phenotypic differences among genetically distant MTBC strains. Indeed, variable transcriptional profiles of genes involved in the lipid metabolic pathways have already been observed among different MTBC strains (Gao et al. 2005). Furthermore, sSNPs within promoter regions or within neighboring open reading frames have been shown to generate new transcriptional start sites that can affect transcription levels of related genes (Rose et al. 2013). A comprehensive system biology approach integrating transcriptomic, proteomic as well as lipidomic data will be required to explore the basis of the various metabolic differences observed across the MTBC phylogeny (Comas and Gagneux 2011).

In conclusion, we present the first study integrating lipidomics and comparative genome sequencing to study MA variability across the phylogeny of MTBC. In addition to substantial strain-specific variability within each MTBC lineage, we observed important lineage-specific patterns linked to specific genomic structures. Given the implications of MA biosynthesis for pathogenesis and immune responses to MTBC, we propose that variable MA patterns contribute to the interaction between different MTBC strains and different human hosts. Moreover, this variation in MA profiles may have important consequences for the current usage and the development of new antibiotics.

## Supporting Information

Figure S1 Mycolic acid profiling of the different MTBC lineages. Box-and-whiskers plot summarizing the representation of various MAs species across the different MTBC lineages; (A) Alpha-MAs, methoxy-MAs and keto-MAs, (B) Methoxy-: keto-MAs (left panel) and oxygenated (e.g. methoxy-MAs plus keto-MAs): Alpha-MAs ratios (right panel), (C) alpha-methyl-*cis*- or *trans*-oxygenated MA species, (D) C26-, C24- and C22-MAs relative abundance (two-tailed Mann–Whitney tests, **P* < 0.05, ***P* < 0.01).

Table S1 Median-fold normalized MA intensities.

Table S2 List of non-synonymous SNPs found in genes involved in MA anabolism.

Table S3 List of MTBC genes demonstrated or suggested to be involved in the biosynthesis of mycolic acid (Takayama et al. 2005; Kastrinsky et al., 2010).
