# Supplementary figures and images for: Lipidomics and genomics of Mycobacterium tuberculosis reveal lineage-specific trends in mycolic acid biosynthesis
Source: Microbiologyopen. 2014 Sep 19;3(6):823–35. doi: 10.1002/mbo3.193 (PMC4263507; doi:10.1002/mbo3.193)

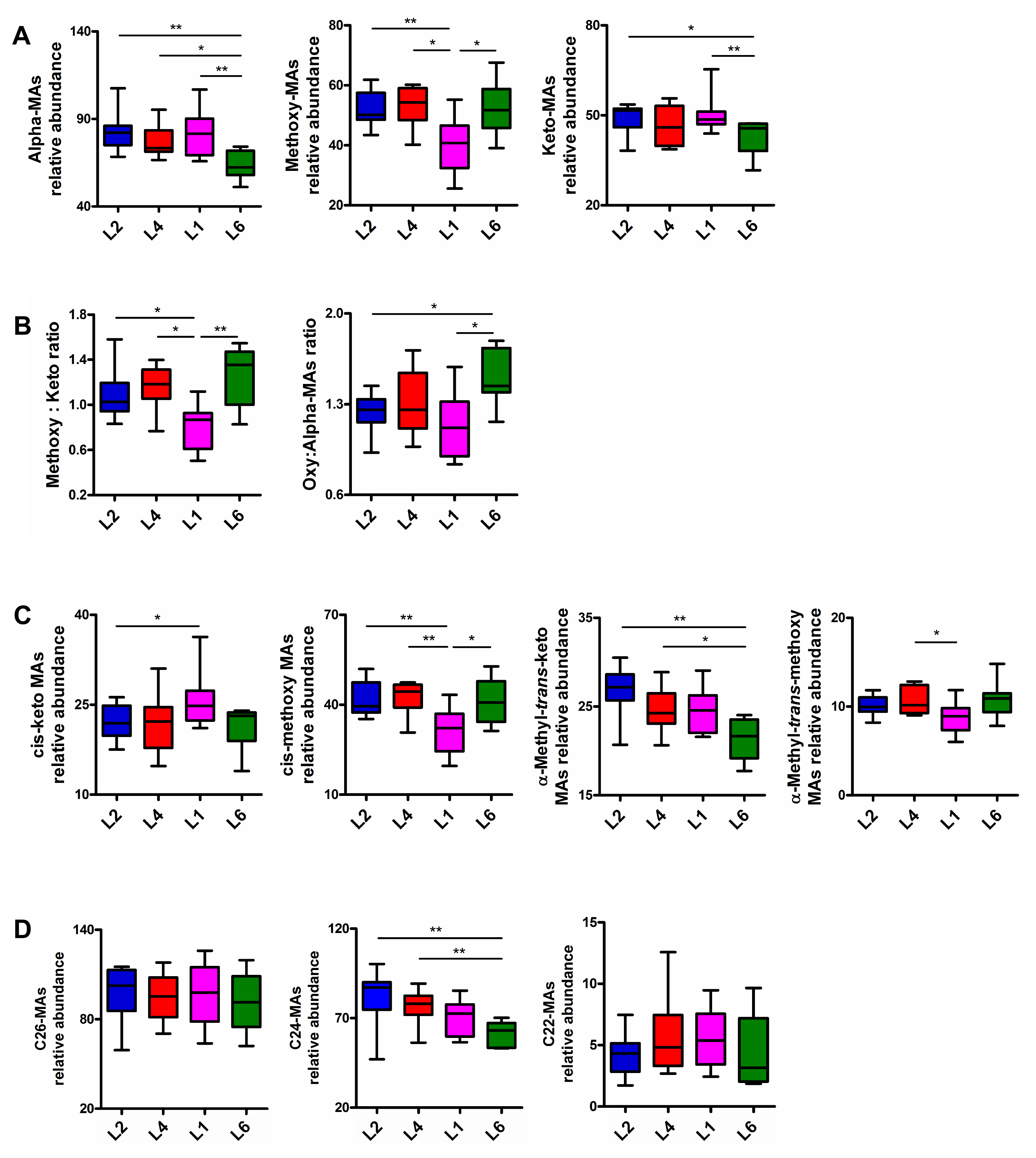

Supplement: Figure S1 — Mycolic acid profiling of the different MTBC lineages. Box-and-whiskers plot summarizing the representation of various MAs species across the different MTBC lineages; (A) Alpha-MAs, methoxy-MAs and keto-MAs, (B) Methoxy-: keto-MAs (left panel) and oxygenated (e.g. methoxy-MAs plus keto-MAs): Alpha-MAs ratios (right panel), (C) alpha-methyl-cis- or trans-oxygenated MA species, (D) C26-, C24- and C22-MAs relative abundance (two-tailed Mann–Whitney tests, *P < 0.05, **P < 0.01). [file mbo30003-0823-sd1.png]

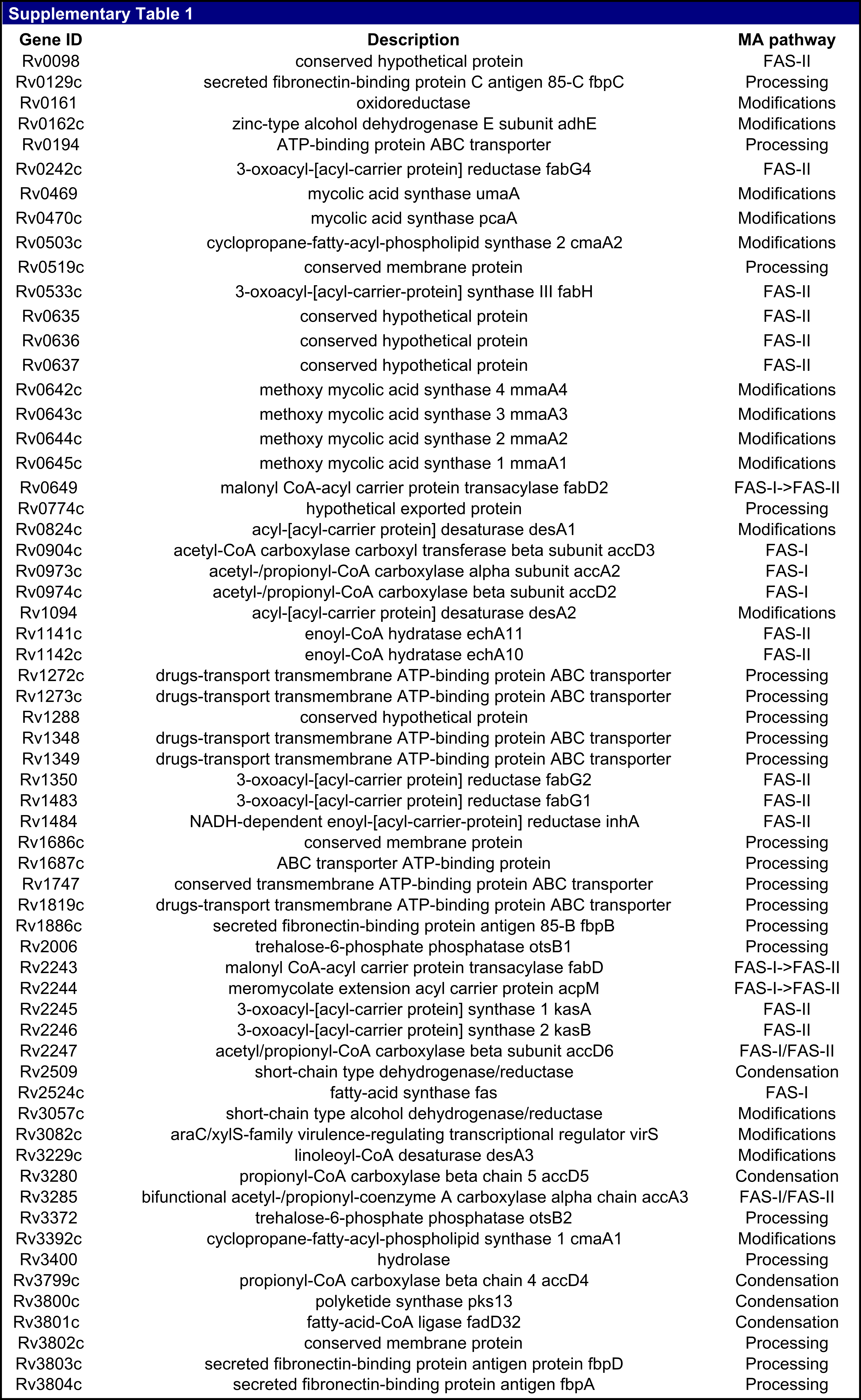

Supplement: Table S3 — List of MTBC genes demonstrated or suggested to be involved in the biosynthesis of mycolic acid (Takayama et al. 2005; Kastrinsky et al., 2010). [file mbo30003-0823-sd4.png]
